# Reducing Setup and Turnover Times in the OR With an Innovative Sterilization Container: Implications for the COVID-19 Era Military Medicine

**DOI:** 10.1093/milmed/usab214

**Published:** 2021-09-01

**Authors:** David F Bradley, Kenneth Romito, James Dockery, Lance Taylor, Nicholas ONeel, Jose Rodriguez, Laura A Talbot

**Affiliations:** Uniformed Services University of the Health Sciences, Graduate School of Nursing, Bethesda, MD 20814, USA; Department of Perioperative Services, Tripler Army Medical Center, Honolulu, HI 96859, USA; Department of Perioperative Services, Brooke Army Medical Center, JBSA-Fort Sam Houston, TX 78234, USA; Department of Perioperative Services, Defense Health Agency, Falls Church, VA 22042, USA; Department of Perioperative Services, Brooke Army Medical Center, JBSA-Fort Sam Houston, TX 78234, USA; Uniformed Services University of the Health Sciences, Graduate School of Nursing, Bethesda, MD 20814, USA; Department of Neurology, University of Tennessee Health Science Center, College of Medicine, Memphis, TN 38163, USA

## Abstract

**Introduction:**

The global 2019 coronavirus pandemic (COVID-19) is setting unprecedented demands on the nation and the military and surgical services. Surgical demands include a large backlog of surgical cases, strain on available resources, and the need for additional measures to prevent exposure. The purpose of this project was to evaluate the feasibility, duration, adverse events, and potential gains associated with using a Turbett Sterilization Pod (TSP) for total joint replacements.

**Materials and Methods:**

A multidisciplinary team used the Plan–Do–Study–Act model to guide this project. A time–motion study was completed in the operating room (OR) to measure the average time required to set up surgical instrumentation for total joint replacement cases that required 12 or more instrument trays. We compared the amount of time it took to complete the setting up of instrumentation using the traditional method versus the TSP method. The traditional method consisted of unwrapping each surgical tray, checking for holes in the blue wrapper, and placing the tray on the back table. In the case of the TSP, the door of the pod was opened, and the instrument trays were transferred directly to the back table. We measured the time the staff took to perform the task using each of these methods.

**Results:**

When compared to the traditional method, the use of the TSP resulted in improved turnover time, decreased room setup time, reduced environmental waste, and eliminated both the effect of damage to wrappers and the time previously spent wrapping surgical trays.

**Conclusion:**

The TSP minimizes the time needed by the staff to set up an OR suite for a total joint replacement, therefore permitting them to focus more on direct patient care. This time improvement suggests that all surgical specialties, including those requiring greater than 12 traditional instrument sets, may experience reduced turnover time between cases. The use of the TSP is one means to help rectify the OR backlog brought on by COVID-19.

## INTRODUCTION

The United States is in the midst of a global 2019 coronavirus pandemic (COVID-19), which is putting unprecedented demands on the nation and the military. The best practice models in the operating room (OR) are continuously evolving as COVID-19 develops.^1^ As the pandemic continues, the ability to predict the progression of COVID-19 cases remains uncertain. The initial recommendation by the DoD on surgical services was to operate only on urgent cases and delay elective surgeries, such as orthopedics, matching Centers for Disease Control and Prevention recommendations.^2^ This created many challenges in the perioperative arena, including a large backlog of surgical cases, causing a strain on available resources and requiring staff to take extra steps to prevent exposure to the virus. This backlog was not unique to the military. According to a global assessment, 73% of surgical procedures would be canceled or postponed.^3^ It was also estimated that if countries increased their normal case load by 20%, it would take approximately 45 weeks to reach pre-COVID-19 workloads and clear backlogs.^3^ Another study projected pre-pandemic caseloads levels would not be enough to clear the newly formed backlog, and if an increase in productivity is not achieved, the backlog will never clear.^4^

As military treatment facilities (MTFs) began to reopen, the realization dawned that mechanisms must be developed to address the overflowing case demand and backlog of surgeries and procedures while maintaining the focus on safety for both patients and staff. In contemplating the clearing of the surgical backlog, the element of time becomes a key factor. While the surgical procedures themselves cannot be rushed, perhaps the preparation time could be optimized to shorten the length of OR room turnovers. Reprocessing and preparation of surgical instruments is a bottleneck for OR efficiency.^5^ We reasoned that improved OR sterilization methods such as the use of sterilization pods would reduce the turnover time between surgical procedures.

Complex surgical cases such as total joint replacements require three or more OR staff members to open 12 or more sets of surgical instruments for each procedure and to place them on a table in the OR for easy access during the procedure. Quality inspection processes for this number of sets often require multiple staff members to streamline the process and prevent delays in first case start times and room turnovers.

The goal of this project was to evaluate the use of an instrument sterilization pod, specifically the Turbett Sterilization Pod (TSP; Turbett Surgical, Rochester, NY), in reducing the amount of time required to open total joint surgical sets and place them on the back table, as compared to the traditional unwrapping method. This project further aimed to reduce turnover times between surgical cases and decrease environmental burden through waste reduction, thus allowing for increased case volume per room per day. The purpose of this project was to evaluate the feasibility, duration, adverse events, and potential gains associated with using a TSP for total joint replacements and to consider the potential impact on these and other surgical procedures during the current COVID-19 pandemic and thereafter.

## METHODS

A multidisciplinary team used the Plan–Do–Study–Act model to guide this project.^6^ A time–motion evaluation was used in the OR to assess the average time to open 12 or more sets for total joint cases. The project was categorized as process improvement by the Brooke Army Medical Center (BAMC) Center for Nursing Science & Clinical Inquiry and the Standardization Committee and determined to not constitute human research.

### Setting

This project was performed at BAMC, a joint military medical center in San Antonio, TX, which is staffed by the military, government civilians, and contract company workers. The facility is the only multidisciplinary military Level 1 trauma center in the country and the largest medical center in the DoD enterprise, averaging 65-70 cases per day. The Department of Operative Services is made up of the OR and the Sterile Processing & Distribution (SPD) section. The OR has 28 rooms spread between two buildings, the main nursing tower (main side, 15 rooms), and a consolidated trauma tower (COTO, 13 rooms). All the total joint procedures were performed on the COTO side with three ORs set aside for the service on each of 3 days per week. The orthopedic service averages two to three cases per day in each of those rooms, with the total averaging 32-34 cases per month. When the staff gather the instrumentation for total joint procedures, an average of 10/15 sets are from vendor companies. Case carts for the total joint procedures are transported via robot to the COTO side. A second MTF was evaluated for comparison, Tripler Army Medical Center (TAMC).

### Initial Planning Phase

The initial phase of preparation and planning was developed by the deputy chief of SPD to decrease room turnover times, increase daily first-case on-time starts, and reduce instrument set defects using a sterilization container in place of traditional wrapping for the instrument sets before autoclaving. The plan was to pilot the use of the container in a specific surgical service and type of surgery, in this case orthopedic service total joint replacement surgery, and determine whether the container could achieve these goals. To simplify the interpretation of the data, the total joint surgery was selected on the basis of its low variability of required instrument sets. The TSP was chosen as a test system based on the ability of its manufacturer to list its consumable filters under the defense agency pricing agreement, thereby enabling the investigators to evaluate its use in a military medical facility. The TSP was also evaluated against the Association of periOperative Registered Nurses (AORN)’s *Guidelines for Sterilization Packing Systems* as being able to safely store and transport surgical instruments during reprocessing.^7^ The evaluation team was assembled from orthopedic total joint program staff surgeons, clinical nurse specialists, representatives from SPD and OR department leadership, and an infection control specialist recruited to provide tracing support for any potential post-op surgical site infections arising during the study duration. The project was submitted to the hospital standardization committee for approval before starting the evaluation.

### Pre-implementation Phase

With the known parameters for weight (375 pounds) and number of instrument sets (15 sets), the surgical team, vendors, and SPD department staff standardized the sets to be supported by each pod. These sets include all of the standard items necessary to complete a primary total joint replacement procedure. Any items not consistent with the pod’s capacity parameters would be processed in the traditional manner with either sterilization pan or wrapping. Sterilizers were evaluated as being compatible with the TSP as recommended by the AORN.^8^ The proper loading of the pods with instrument sets was checked by both the vendor and SPD department staff before sealing and locking the unit. The pod was sterilized in the SPD facility, and SPD personnel transported the loaded and sterilized pod to the OR.

### Implementation

**FIGURE 1.**
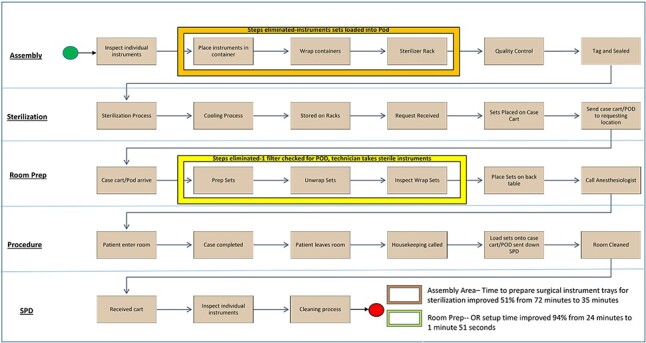
BAMC SPD instrument sterilization process time reduction using Turbett sterilization process. BAMC, Brooke Army Medical Center; SPD, Sterile Processing & Distribution.

The 30-day evaluation of the surgical pod at BAMC involved three orthopedic surgeons assigned to the total joint service for total knee and total hip procedures (June 11, 2019, to July 9, 2019). Three registered nurses, five OR surgical technicians, and the SPD staff received individual training on the surgical sterilization pod before the start of the trial. To ensure that the surgical pod and its contents would meet all of the Association for the Advancement of Medical Instrumentation ST 79 guidelines for sterilization within our facility,^9^ a trial of six test loads were sterilized as per the manufacturer’s instructions, while varying the time and temperature within accepted parameters. A workflow analyst monitored each step of the process to track processing times of both the current procedure and the pod method (Fig. 1). The evaluation was initially projected to include at least 72 total joint surgical procedures, based on historical scheduling data within the facility. Due to electrical issues that resulted in the closure of half of the ORs within the facility and consequently a decrease in scheduled total joint cases, the inclusion criteria were modified to include other orthopedic cases. The final count of surgical procedures using the sterilization pod in the trial was eight cases. For the traditional method, similar OR cases were selected using the Surgery Scheduling System (S3) from September 3, 2019, to October 8, 2019. A total of 10 traditional orthopedic cases were selected.

### Traditional Method

In the SPD, instrument sets were assembled, placed into a metal pan or wrapped in blue wrap and secured with a heat-sensitive tape, loaded into a sterilization rack, and placed into the sterilizer. After the sterilizer had completed its cycle, it was opened, and the racks and instruments were allowed to cool. The sets were then placed in their proper storage areas until use. For the total joint procedure, the instrument sets specified by the surgeon’s preference card were retrieved by SPD staff, placed on a case cart, and transported to the OR by a robotic system. The transportation process was timed to take 18 minutes. Once the sets arrived in the OR, they were opened, inspected by the circulator nurse, and placed onto the back table by the surgical technician. A typical total joint case uses 12 or more sets of instruments. The traditional method involves unwrapping each set, checking for holes in the blue wrapper, and placing intact sets on the back table. If holes are found, the set is taken out of circulation until it can be re-sterilized for use another time. If enough sets have holes, the procedure may be delayed until sufficient replacement sets have been delivered or cancelled altogether. Holes and waste data were derived from the OR Debrief Tracking Tool.

### Sterilization Container (Pod) Method

The pod sterilization method also began in the SPD. The instrument sets specified by the surgeon’s preference card were assembled, placed into a metal pan, and loaded into the pod. The pod was sealed with one filter sheet in place of traditional surgical wrap and placed into the sterilizer. After the sterilizer completed its cycle, the pod and the enclosed instruments were allowed to cool. The pod and its contents were then transported to the OR by SPD staff, which took 4 minutes. When the pod arrived in the OR, its door was opened, the filter was inspected for the presence of pinholes by the circulator nurse, and the instruments were placed onto the back table by the surgical technician. The 12 or more sets used by a typical total joint case fit into a single pod.

### Outcome Measures

A workflow analyst collected data to evaluate several parameters in the SPD and the OR (Fig. 1). The amount of time SPD staff needed to perform quality checks and prepare instruments (wrapping instrument sets or placing them into the sterilization pod) was measured. Turnover time was measured from the time one patient left the OR until the next patient arrived using timed measurements (traditional method, *n* = 10; pod, *n* = 8). Also assessed were the number of staff members needed to set up the back table and the time it took them to move the instrument sets from the case cart or pod to the back table (Room Setup Time), utilizing the work analyst time (*n* = 1) and the Surgery Scheduling System (S3) data (*n* = 16). Room Setup Time was measured at TAMC comprising six total joint cases per group.

### Statistical Analyses

Descriptive statistics were calculated including frequencies, means, SDs and percentages. T-test statistics were used to determine differences between the traditional method and the pod method. A two-tailed *P*-value of ≤.05 established statistical significance. For the outcome measure of OR setup time in which the sample size was one, the calculation was based on 16 Pod cases with a *t*-score of 29.5 for the traditional method.

## RESULTS

Turnover times using the traditional method (before implementation) averaged 48 minutes (SD = 11). After implementation of the pod method, the turnover time decreased by nearly a third, to 33 minutes (SD = 7; *t* = 3.54; df = 15.45; *P *= .003, Welch two-sample *t*-test). OR setup time improved 94% from 24 minutes to 1 minute 51 seconds (SD = 45 seconds; *t* = 29.5, df = 15, *P* = <.0001). At TAMC, the POD averaged 95 seconds (SD = 25) for room setup, whereas the traditional was 550 seconds (SD = 128) (*t* = −8.5, SD = 5.4, *P* = .0002, *t*-test). The number of man-hours required for SPD personnel to prepare surgical instrument trays for sterilization improved 51% from the traditional method’s 72 minutes to 35 minutes with the pod method. The “show-stopping” adverse event of finding holes in sterilization wrappers, which often led to surgery cancellations, was eliminated with the use of the TSP. The monthly 12 holes-in-wrappers events previously observed with the traditional method were cut to virtually no pinholes seen with the pod method. The use of the TSP reduced delays in first case start times by approximately 15 minutes and reduced the amount of surgical waste from four to five bags to two, a 50%-60% reduction. Figure 1 illustrates the reduction in steps and time from the traditional method to the pod method. To date, zero surgical site infections have been reported when the TSP was used during instrument sterilization.

The ability to customize every surgical pod to each surgeon’s request to ensure that all requested instrumentation was provided to the OR resulted in improved communication between the vendor teams and SPD staff. The removal of excess surgical instrumentation decreased the workload and costs involved in processing and sterilization. The sterilization pod was also effective in support of emergent trauma surgical cases. Its utilization decreased delays associated with the assembly and processing of requested vendor sets, which were processed with minimal impact on current workload.

## DISCUSSION

This time–motion study, which evaluated its impact pre-operatively, showed a significant decrease in turnover time and setup time between surgical cases with the pod when compared to the traditional method. The outcomes obtained from this initiative support several key concerns impacting surgery during a global pandemic and possibly future crisis. Due to COVID-19, elective surgery backlogs are increasing, and measures must be taken to address access to surgical care. The time saved during OR turnover and back table setup using the TSP maximizes OR utilization and increases access to care (the possibility that OR leadership may be able to add an extra case at the end of the day).

In addition, shortages of nursing staff in the United States have resulted in limited human resources to sustain OR operations safely and efficiently. By introducing the TSP to the OR workflow, fewer manpower hours are needed for completing tasks while setting up the back table, which enables better utilization of human resources during this pandemic. Because OR clinical labor represent a significant portion of the surgical services budget, the overall costs would be reduced.^10^

Finally, the integration of new technology, such as the TSP, contributes to productivity and efficiency in the OR. We observed that the use of the TSP resulted in decreased OR setup time, reduced surgical waste, and eliminated both the effect of damage to wrappers and the time previously spent wrapping surgical instrument sets. The disposal of medical waste can account for up to 20% of a hospital’s environmental services budget.^11^ An average of 15 instrument wraps and no-drip padding per case was replaced by 1 filter and 6 plastic locks, further promoting the facility’s Go-Green initiatives and smaller footprint. A nationwide shortage of sterilization wrap has prompted the AORN to issue a safety statement with alternative sterilization approaches such as the POD.^12^

### Limitations of the POD

One limitation of the use of the sterilization pod was its large size, which caused a storage issue. Additionally, because the pods did not fit in the robotic case cart transport system at BAMC, extra SPD staff members were required to manually transport the sterilized pods from the SPD to the COTO ORs. Extra tasking and deployments of military staff members can affect the number of individuals at any given time who are available to transport the pods. Finally, the cost and procurement process involved in purchasing the TSP can be daunting. The military purchasing process can take up to a year on high dollar items and justifications, and data must show a significant return on investment or an improvement in quality and safety before the purchase will be approved.

### Study Limitations

This quality improvement project evaluated orthopedic total joint replacement surgical cases in two large MTFs. The amount of time improvements may not be applicable to other MTFs or surgical procedures. This project focused on only one orthopedic procedure. This preliminary work consisted of combining several data sets, including the data from an independent analyst. Future research is needed using other surgical procedures, a larger sample size, additional MTFs, and measuring cost savings related to decreased workload in processing and sterilization.

## CONCLUSIONS

The backlog of elective surgeries associated with COVID-19 in MTFs is unprecedented.^13^ Consideration must be given to the possibility of viral contamination to staff during surgery. The TSP can be used for any surgical specialty that requires many sets and reduces the exposure time and staff. The TSP minimizes the time spent on OR setup and enables the OR staff to focus more on direct patient care (for example, assisting anesthesia during induction or providing patient comfort measures). Fewer nurses or techs are needed for case setup, thereby reducing exposure to COVID-positive patients. The faster setup of the room translates to reduced time patients must remain under anesthesia. The time saved by using the TSP can also be used to add backlog cases to the surgical schedule for the day. Because the need to wrap surgical instruments is eliminated with the TSP, SPD staff have more time to check for bioburden and perform quality checks on instrument sets and less surgical waste is produced, which in turn decreased the need for its removal. The pod can also house (in one place) the instruments needed for COVID-19 patient emergencies, potentially allowing damage control surgery to be started faster.

## References

[1] Al-Jabir A , et al: Impact of the coronavirus (COVID-19) pandemic on surgical practice - part 1. Int J Surg (London, England) 2020; 79: 168–79. 10.1016/j.ijsu.2020.05.022 PMC7214340 32407799

[2] Centers for Medicare & Medicaid Services : Centers for Medicare and Medicaid Services (CMS) recommendations: re-opening facilities to provide non-emergent non-COVID-19 healthcare: phase 1. Available at https://www.cms.gov/files/documents/covidflexibility-reopen-essential-non-covid-services.pdf; accessed October 23, 2020.

[3] COVIDSurg Collaborative : Elective surgery cancellations due to the COVID-19 pandemic: global predictive modelling to inform surgical recovery plans. Br J Surg 2020; 107(11): 1440–1449.doi: 10.1002/bjs.11746 32395848 PMC7272903

[4] Salenger R , et al: The surge after the surge: cardiac surgery post-COVID-19. Ann Thorac Surg 2020; 110(6): 2020–2025.doi: 10.1016/j.athoracsur.2020.04.018 32376350 PMC7196543

[5] Coban E : The effect of multiple operating room scheduling on the sterilization schedule of reusable medical devices. Comput Ind Eng 2020; 147.doi: 10.1016/j.cie.2020.106618

[6] Silver SA , et al: How to begin a quality improvement project. CJASN 2016; 11(5): 893–900. 27016497 10.2215/CJN.11491015 PMC4858490

[7] Association of periOperative Registered Nurses(AORN) :Guidelines for sterilization packaging systems.*Guidelines for Perioperative Practice*. AORN; 2021:985–1013.

[8] Croke L : Guideline for sterilization packing systems. Vol 110. AORN Periop Briefing, 2019; 110(3): P8–10. 10.1002/aorn.12813 31465569

[9] Association for the Advancement of Medical Instrumentation . *ST 79*. AAMI; 2019.

[10] Biala GE , Fitzpatrick TA: *Healthcare Executive’s Guide to Navigating the Surgical Suite*. Sigma Theta Tau International; 2019.

[11] Conrardy J , et al: Reducing medical waste. AORN J 2010; 91(6): 711–21. 20510944 10.1016/j.aorn.2009.12.029

[12] Association of periOperative Registered Nurses (AORN) : Safety statement from AORN. Available at https://www.aorn.org/about-aorn/aorn-newsroom/sterilization-wrap; accessed April 28, 2021.

[13] Brandman DM , et al: Modelling the backlog of COVID-19 cases for a surgical group. Can J Surg 2020; 63(5): E391–2. 32856888 10.1503/cjs.011420 PMC7608701

